# Comparative Transcriptomic Analysis Reveals Molecular Profiles of Central Nervous System in Maternal Diapause Induction of *Locusta migratoria*

**DOI:** 10.1534/g3.119.400475

**Published:** 2019-08-12

**Authors:** Aftab Raza Jarwar, Kun Hao, Ellyn Valery Bitume, Hidayat Ullah, Dongnan Cui, Xiangqun Nong, Guangjun Wang, Xiongbing Tu, Zehua Zhang

**Affiliations:** * State Key Laboratory for Biology of Plant Diseases and Insect Pests, Institute of Plant Protection, Chinese Academy of Agricultural Sciences, Beijing 100193, People’s Republic of China,; † Institute of Pacific Islands Forestry, Pacific Southwest Research Station, USDA Forest Service, Volcano, Hawaii and; ‡ Department of Agriculture, The University of Swabi, Anbar 23561, Swabi, Khyber Pakhtunkhwa, Pakistan

**Keywords:** CNS, FOXO signaling pathway, Maternal effect, Photoperiod, *Lm-takeout*

## Abstract

Egg diapause in *Locusta migratoria* L. (Orthoptera: Acridoidea) is believed to be influenced by maternal photoperiod. However, the molecular mechanism regulating the phenomenon of maternal diapause induction is unclear. Here we performed transcriptomic analyses from the central nervous system (CNS) of migratory locusts under long and short photoperiods to identify differentially expressed genes (DEGs) related to diapause induction. There were total of 165750 unigenes from 569491 transcripts, and 610 DEGs were obtained in S_CNS (CNS of short photoperiod treated locusts) *vs.* L_CNS (CNS of long photoperiod treated locusts). Of these, 360 were up-regulated, 250 were down-regulated, and 84 DEGs were found to be related to FOXO signaling pathways, including citrate cycle/TCA cycle, glycolysis/ gluconeogenesis, oxidative phosphorylation, and PI3K-Akt. The qRT-PCR validation of mRNA expression of 12 randomly selected DEGs showed consistency with transcriptome analysis. Furthermore, the *takeout* gene thought to be involved in circadian rhythm was cloned and used for RNAi to observe its function in maternal diapause induction. We found that the mRNA level of *Lm-takeout* was significantly lower in ds*takeout* treatments as compared to the control under both long and short photoperiods. Similarly, the offspring diapause rate was significantly higher in ds*takeout* treatment as compared to the control only in short photoperiod. This shows that the *Lm-takeout* gene might be involved in the inhibition of maternal diapause induction of *L. migratoria* under short photoperiods. The present study provides extensive data of the CNS transcriptome and particular insights into the molecular mechanisms of maternal effects on egg diapause of *L. migratoria*. As well for the future, the researchers can explore other factors and genes that may promote diapause in insect species.

Diapause is an adaption for the survival of insects during the inimical season, a well-known phenomenon in many species of insects that may occur at any developmental stage from embryo to adult (Masaki 1980). Various factors, such as temperature, photoperiod, drought, food shortage, and hormonal response can influence diapause induction (Chippendale 1982). Among them, photoperiod is considered one of the most important environmental factors that affect the probability of maternal regulation of offspring diapause (Mousseau 1991). This phenomenon may occur during ovary development, when the signals of temperature and light are received through central nervous system (CNS), and subsequently transferred to the offspring (Emerson *et al.* 2009; Sato *et al.* 2014; Yamashita *et al.* 2001). In the progeny of silkworm, *Bombyx mori*, environmental temperature and photoperiod during parental embryonic development influences diapause (Sato *et al.* 2014). Similarly, offspring diapause is also reported in blowfly, *Calliphora vicina*, (Vinogradova 1974). On the other hand, circadian clocks are also involved in measuring the length of days and nights to regulate photoperiodic diapause in the bean bug, *Riptortus pedestris* (Saunders 2002; Ikeno *et al.* 2010; Koštál 2011).

Many genes and pathways have been linked to photoperiodic diapause regulation (Denlinger 2002; Bao and Xu 2011). Diapause is shown to be regulated by the FOXO signaling pathway, which is a downstream pathway regulated by circadian clocks in mosquitos (Sim *et al.* 2015). In the FOXO signaling pathway, insulin can also regulate the diapause process through the synthesis of juvenile hormones (Sim and Denlinger 2008). The CNS and brain influence the response of the circadian clock in dopamine treated larval diapause of drosophilid fly, *Chymomyza costata*, and cotton bollworm, *Helicoverpa armigera*, (Košt’ál *et al.* 2000; Zhang *et al.* 2013). Involvement of the circadian oscillator photoperiodic time measurement in diapause was first proposed in plants and subsequently by many other organisms including vertebrates (Daan and Aschoff 1982). The circadian clock genes *per* and *cyc* have been reported to be involved in ovarian development during varying photoperiodic conditions in *Riptortus pedestris* (Bradshaw and Holzapfel 2010). *Takeout* is a circadian output pathway gene also involved in regulation of energy metabolism and behavior in insect species (Sarov-Blat *et al.* 2000). A previous study on CHIP-seq analysis revealed that *takeout* played an important role in photoperiodic diapause of *Culex pipiens* (Sim *et al.* 2015). Our previous study showed that FOXO plays an important role in regulating offspring diapause in *L. migratoria* (Hao *et al.* 2019). However, no such evidence has been previously confirmed that there was any relationship between *takeout* and FOXO signaling pathway.

The migratory locust, *Locusta migratoria* L. is an important agricultural pest commonly distributed throughout the world (Uvarov 1966; Uvarov 1977; Farrow and Colless 1980; Chen 1999). Maternal temperature and photoperiod are effective determinants of the transgenerational egg diapause of *L. migratoria* (Tanaka 1994). Our previous study demonstrated that insect hormone biosynthesis, the insulin signaling pathway, and the peroxisome proliferator-activated receptor (PPAR) signaling pathway are involved in diapause regulation of *L. migratoria* eggs (Hao *et al.* 2017). Similarly, our transcriptomic study on ovaries and fat bodies in diapause induction of maternal *L. migratoria* also revealed the candidate genes related to the FOXO pathway and verified their diapause regulation functions (Hao *et al.* 2019). However, such mechanisms in CNS of maternal migratory locusts are still unknown. In this study, we analyzed the transcriptomes from CNS of adult females reared under long and short photoperiodic conditions and identified the significantly differentially expressed genes (DEGs) that are related to the locust’s offspring diapause. We further investigated the function of *Lm-takeout* gene on diapause regulation by using RNAi. Our results can improve our understanding of diapause-associated functions of candidate genes and reveal molecular mechanisms of maternal photoperiod effect on offspring diapause.

## Materials and Methods

### Insect rearing and tissue collection

The colony of *L. migratoria* was originally collected from the field at Tianjin, China (38°49’N, 117°18’E), maintained at the State Key Laboratory for Biology of Plant Diseases and Insect Pests, Institute of Plant Protection, Chinese Academy of Agricultural Sciences, Beijing. Mesh cages of 20 × 20 × 28 cm were used for rearing locusts. Fresh wheat seedlings were fed to the locusts on daily basis. Second instar locusts were transferred into artifical climate boxes (PRX-250B-30, Haishu Saifu Experimental Instrument Factory, Ningbo, China), which were used in two different photoperiod cycles for long and short day length. The photoperiodic condition used for non-diapause locusts in the experiment was 16:8 L:D, 27° and 60% relative humidity (RH). To induce diapause, temperature and RH remained the same while day length was reduced to 10:14 L:D (Hao *et al.* 2019). The potential effect of circadian cycles when tissues were collected at same circadian cycles, *i.e.*, at 2 hr lights off (short day) and 2 hr lights on (long day) as per the procedure of Ren *et al.* (2018). The central nervous system (CNS), which includes the brain, spinal cord, and a complex network of neurons, was separately collected from female individuals (72 hr after matured female adults). Three biological replicates were conducted for each treatment. Tissues were dissected into cold RNase-free saline solution. Each tissue had three replicates from individual treatments collected in both long and short photoperiods. All samples were quickly frozen in liquid nitrogen and then kept at -80° until RNA extraction.

### RNA extraction and RNA-seq

A total of six individual females of *L. migratoria* were used for RNA extraction independently by applying Trizol followed by library construction and sequencing using the previously reported protocol (Grabherr *et al.* 2011). Total RNA was isolated from collected CNS tissues by using the RNeasy Plant Mini kit with column DNase digestion (Qiagen, Hilden, Germany) following the manufacturer’s instructions. RNA concentration was then measured using Qubit RNA Assay Kit in Qubit 2.0 Flurometer (Life Technologies, Carlsbad, CA, USA). Additionally, RNA integrity was assessed using the RNA Nano 6000 Assay Kit of the Bioanalyzer 2100 System (Agilent Technologies, Santa Clara, CA, USA). All six of the samples with RNA integrity number (RIN) values above 8 were used for construction of the libraries. Sequencing libraries were generated using NEBNext Ultra RNA Library Prep Kit for Illumina (NEB, USA) following manufacturer’s recommendations, whereas index codes were added to attribute sequences to each sample. Finally, PCR products were purified (AMPure XP system) and library quality was assessed using an Agilent Bioanalyzer 2100 system. The clustering of the index-coded samples was performed on a cBot Cluster Generation System using TruSeq PE Cluster Kit v3-cBot-HS (Illumina) according to the manufacturer’s instructions. After cluster generation, the library preparations were sequenced on an Illumina Hiseq 2500 platform and 125 bp paired-end reads were generated.

### Sequence assembly, annotation and DEGs analysis

Sequencing data of CNS samples were analyzed independently. Raw data were processed into clean data reads, whereas the reads containing adapters, poly-N, and those of substandard quality were removed. Transcriptome assembly was achieved based on the clean data using Trinity with min kmer cov set to default value of 2 and all other parameters set to default (Grabherr *et al.* 2011). Gene function was annotated based on the following seven databases: Nr (NCBI non-redundant protein sequences), Nt (NCBI nucleotide sequences), Pfam (Protein family), KOG/COG (Clusters of Orthologous Groups of proteins), Swiss-Prot (a manually annotated and reviewed protein sequence database), KEGG (Kyoto Encyclopedia of Genes and Genomes) and GO (Gene Ontology). Data for each sequenced library was analyzed using BLAST (Basic Local Alignment Search Tool) with a cut-off *e*-value of 10^−5^. Prior to differential gene expression analysis, for each sequenced library the read counts were adjusted using edge R program package through one scaling normalized factor. Differential expression analyses of two samples were performed using the DEGs seq (2010) R package (1.10.1). The *p*-value was adjusted using *q*-value (Young *et al.* 2010). Whereas *q*-value < 0.005 & |log_2_ ratio (fold change) | > 1 was specified as the threshold for significantly differential expression.

### cDNA synthesis and qRT-PCR

Total RNA collected from CNS under long photoperiod (L_CNS1, L_CNS2 and L_CNS3 mixed together) and short photoperiod (S_CNS1, S_CNS2 and S_CNS3 mixed together) was used for further investigation. cDNA was synthesized from the mixed RNA samples by using M-MLV reverse transcriptase (Life Technologies GmbH, Darmstadt, Germany) and recombinant RNase inhibitor (TaKaRa Biomedical Technology Co. Ltd., Beijing, China). The expression levels of 12 DEGs in CNS tissues were determined by qRT-PCR using SYBR Premix Ex Taq (TaKaRa Dalian, China) in an ABI 7500 real-time PCR system as per the manufacturer’s instructions (Applied Biosystems, Foster City, CA, USA). qRT-PCR was performed in the following conditions: 95° for 10 min; 40 cycles of 95° for 15 s, 60° for 45 s. Gene expression was quantified using 2^−ΔΔCt^ method (Livak and Schmittgen 2001), with elongation factor 1 (*ef-1*) as the internal control for normalization of data using specific primers for qRT-PCR (Table S1).

### RNA interference

RiboMAXT^M^ System-T7 (Promega, Madison, WI, USA) was used to generate dsRNA by *in vitro* transcription following the manufacturer’s instructions. Templates for *in vitro* transcription reactions were prepared by PCR amplification from plasmid DNA of the cDNA clone of target gene (*Lm-takeout*) using the primer pairs with T7 polymerase promoter sequence at 5′-end (Table S1). The length of *Lm-takeout* was 532 bp. A total of 5μl of dsRNAs (2 μg/μL) for the target gene (*Lm-takeout*) and ddH_2_O water as a control were injected between the 2^nd^ and 3^rd^ ventral part of the abdominal segment of adult females within 72 hr of molting. A total of 75 females were injected and divided into three groups. The effects of RNAi on the mRNA levels were investigated by qRT-PCR 48 hr after injection. To quantify transcript levels of *Lm-takeout*, total RNA was extracted from the entire body of the locust. For each target gene, three individuals from each treatment under both long and short photoperiods were used for RNA extraction.

### Diapause rate detection

Locusts were moved to new mesh cages and provided with fresh wheat seedlings as described above. Meanwhile, males were introduced to treated virgin adult females in each replicate. The bottom of the cages was covered with a 2 cm layer of sieved sterile sand and maintained until the locusts began to lay eggs. Once oviposition was observed, eggs were collected every 48 hr for 10 days using a camel paint brush and transferred into plastic Petri dishes (90 mm × 50 mm). The eggs were then incubated on vermiculite with 20–30% water content before being transferred to 27° and 60% RH to slowdown development. We obtained approximately 150 eggs from 2-3 pods, which were then used in each experimental replication. Eggs were kept under 27° for 30 days until eclosion of 1^st^ instar nymphs ceased (^1^H). To account for non-viable eggs, all remaining unhatched eggs were kept at 4° for 60 days to receive sufficient time to break the diapause at 27° for 30 days and any further 1^st^ instar emergence recording (H2). The diapause rate (DR) was calculated as:

$$\text{DR}(\text{\%})=\frac{\text{H}2}{\text{H}1+\text{H}2}\times 100\text{\%}$$

### Statistical analysis

Differences between treatments were compared by using a Student’s *t*-test. Differences were considered significant at *P* < 0.05. Values are presented as mean ± SE. Data were analyzed using SPSS software (version 15.0; SPSS Inc., Chicago, IL, USA) and figures were constructed using GraphPad Prism software (version 6.01; GraphPad Software Inc., San Diego, CA, USA).

### Data availability

All the raw reads data files have been deposited at the NCBI Sequence Read Archive database under project number PRJNA392007 (https://www.ncbi.nlm.nih.gov/bioproject/PRJNA392192); BioSample number as L-CNS1: SAMN07280117, L-CNS2: SAMN07280118, L-CNS3: SAMN07280119, S-CNS1: SAMN07280120, S-CNS2: SAMN07280121, S-CNS3: SAMN07280122 and SRA number as SRR5759362, SRR5759363, SRR5759364, SRR5759365, SRR5759366, SRR5759367. The supplemental files submitted to https://gsajournals.figshare.com contain the following data; Table S1 List of specific primers used for qRT-PCR in the current study. Table S2 S_CNS *vs.* L_CNS DEGs annotated into KEGG pathways. Table S3 S_CNS *vs.* L_CNS DEGs GO enrichment classification. Table S4 S_CNS *vs.* L_CNS DEGs annotations of key genes related to diapause induction. Supplemental material available at FigShare: https://doi.org/10.25387/g3.9250532.

## Results

### Transcriptome sequencing and annotation assembly

Transcriptomes from the CNS of *L. migratoria* under both long and short photoperiods were sequenced and analyzed. Six mRNA libraries were constructed from CNS under long (L_CNS) and short photoperiods (S_CNS) following three biological repeats for each photoperiod. We obtained approximately 65.9-89.1 million clean reads with a Q20 > 95% along with 9.89-13.38 G bases for each replicate (Table 1). The clean RNA-Seq data were deposited in the NCBI Sequence Read Archive (SRA) database under accession numbers **SRR5759362**, **SRR5759363**, **SRR5759364**, **SRR5759365**, **SRR5759366** and **SRR5759367**. A total of 569491 transcripts including 165750 unigenes were assembled (Table 1). The molecular mechanism of important transcriptomic profiles and gene functions were deduced after the elimination of the repeated and short-length sequences. Approximately 165750 non-redundant unigenes were screened for similarity in seven public databases *i.e.*, Nr, Nt, Swiss-Prot, KEGG, GO, COG and Pfam (*e*-value < 0.00001). The annotation results showed that 44340 unigenes (26.75%) matched in the Nr database, whereas 8539 (5.15%) unigenes matched to the Nt database. We found that a smaller percentage of 15.1% (25034 unigenes) was obtained when searching against the Swiss-Prot database rather than against the Nr database. In total, there were 59414 unigenes (35.84%) successfully annotated in at least one of the seven databases with 3584 unigenes (2.16%) in all seven databases (Table 2).

**Table 1:** Summary of RNA-seq metrics from CNS transcriptomes of *Locusta migratoria* under long and short photoperiods

Sample	Clean reads	Clean bases (G)	Q20 (%)	Number of transcripts	Number of unigenes
L_CNS1	88798108	13.32	95.78	569491	165750
L_CNS2	70803344	10.62	95.83		
L_CNS3	89170204	13.38	95.44		
S_CNS1	70245966	10.54	96.14		
S_CNS2	65957458	9.89	96.47		
S_CNS3	70465866	10.57	96.21		

**Table 2:** Blast analysis of non-redundant unigenes in contradiction of public databases

Reference databases	Number of unigenes	Percentage (%)
Annotated in NR	44340	26.75
Annotated in NT	8539	5.15
Annotated in KO	12292	7.41
Annotated in Swiss Prot	25034	15.1
Annotated in PFAM	40094	24.18
Annotated in GO	40230	24.27
Annotated in KOG	13984	8.43
Annotated in all Databases	3584	2.16
Annotated in at least one Database	59414	35.84

### DEGs from CNS of L. migratoria

**Figure 1:**
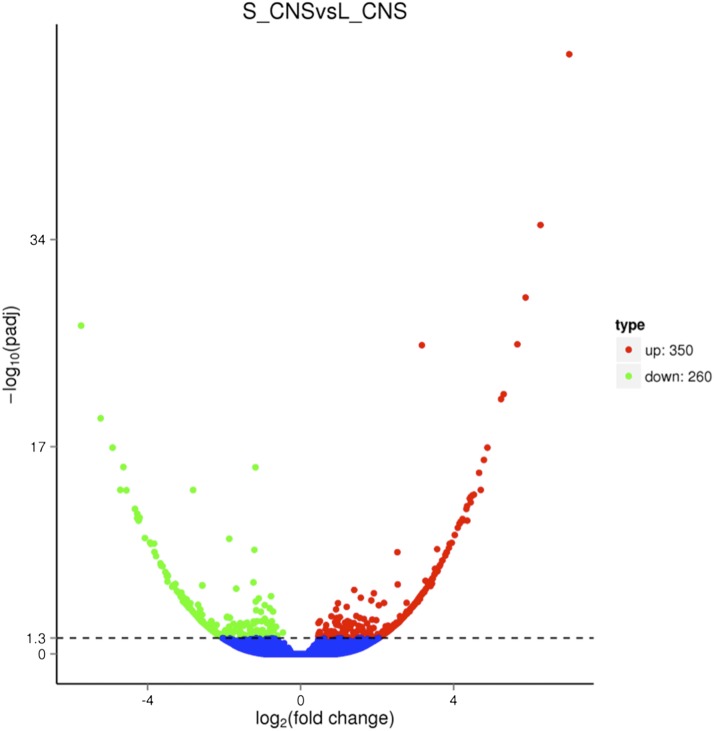
Fold change of DEGs from CNS of *L. migratoria* under long and short photoperiods. The X-axis represents the change of gene expression in different groups; the Y-axis represents the statistical significance of gene expression change. -log_10_ (*p*adj) means -log_10_ (adjusted *p*-value). The smaller the adjusted *p*-value in -log_10_ (*p*adj), the greater the significant difference. Blue dots in the figure represent the genes with no significant difference; red dots represent the up-regulated genes with significant difference; green dots represent the down-regulated genes with significant difference.

DEGs annotations provide valuable resources for further investigation in the study of next generation egg diapause of migratory locusts. A total of 610 DEGs transcriptomes were distinguished in CNS samples under both long and short photoperiods. Among them, 350 were found to be up-regulated and 260 were down-regulated in S_CNS *vs.* L_CNS (Figure 1). These DEGs were annotated into KEGGs pathways to identify the most crucial and significant FOXO related signaling pathway. Results showed that MAPK signaling pathway (ko04010), PI3K-Akt signaling pathway (ko04151), longevity regulating pathway (ko04213), oxidative phosphorylation (ko00190), biosynthesis of secondary metabolites (ko01110), ribosome (ko03010), and glycolysis / gluconeogenesis (ko00010), etc. were among the top annotated pathways (Table S2). DEGs related to oxidative phosphorylation (ko00190), including cytochrome c oxidase (*cox1*, *cox2*), ATPeF0A and NADH dehydrogenase *nd2* were more often found down-regulated in S_CNS than in L_CNS. Whereas, DEGs related to ribosome (ko03010) including RP-l10, RP-l39, RP-L6e, RP-S23e, RP-SAe, RP-S29e and RP-L8e were more often found up-regulated in S_CNS than in L_CNS (Table S2).

### Functional classification by GO enrichment of DEGs

**Figure 2:**
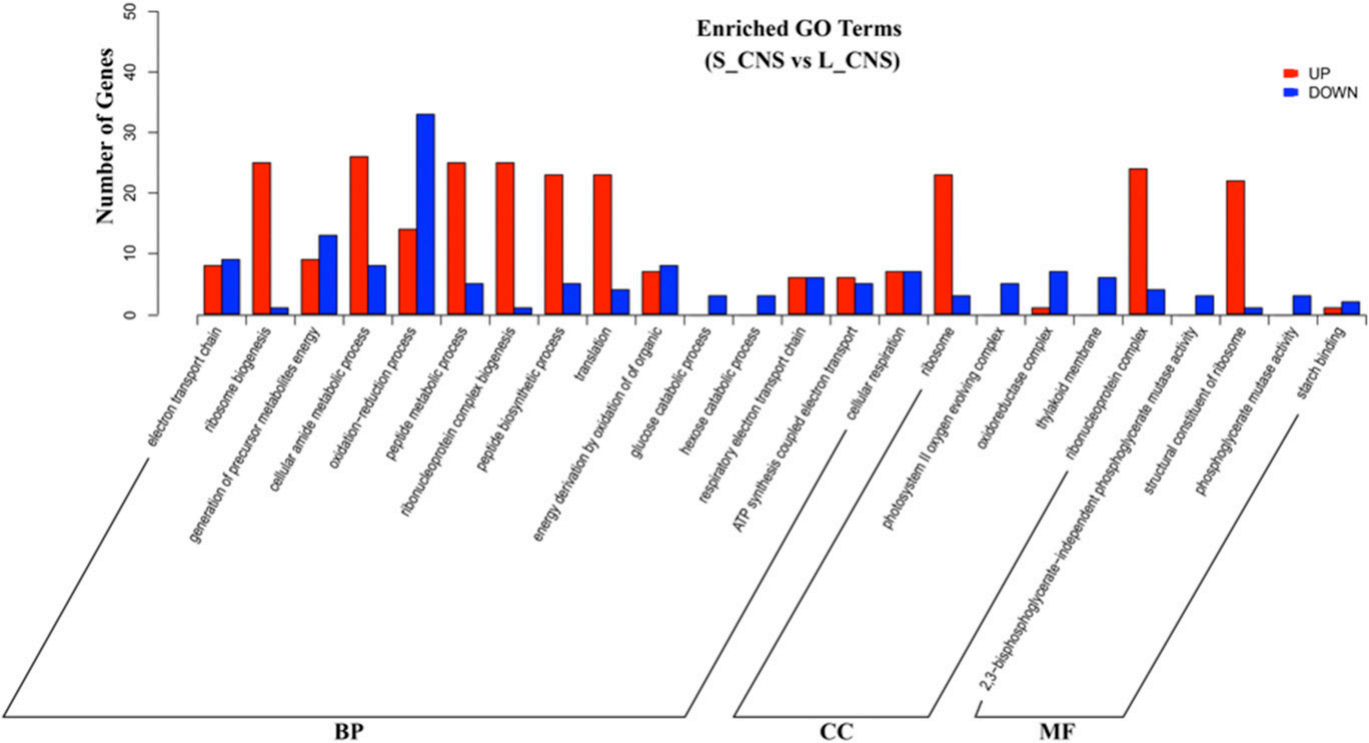
GO enrichment classification of identified gene functions cover with major three classifieds: molecular function (MF), cellular component (CC), and biological process (BP). The X-axis represents the number of DEGs; whereas the Y-axis represents the GO terms. Red columns represent the up-regulated DEGs, whereas blue columns represent the down-regulated DEGs.

A main goal of our study was to reveal the biological functions for further understanding of DEGs involved in maternal diapause induction. Hence, all of the DEGs were mapped to the whole transcriptome background and given GO annotations. GO enrichment (*P* < 0.1) analysis revealed that 506 DEGs in S_CNS *vs.* L_CNS, including 339 up-regulated and 167 down-regulated were subsequently classified into 29 items. Among these, 396 DEGs were mapped to biological processes, 73 were mapped to cellular components, and 37 were mapped to molecular functions. Of the ‘biological process’ related genes, most up-regulated DEGs were involved in ribosome biogenesis, cellular amide metabolic process, peptide metabolic process, ribonucleoprotein complex biogenesis, peptide biosynthetic process, and translation. On the other hand, most of the down-regulated DEGs were involved in the oxidation-reduction process, glucose catabolic process and hexose catabolic process. Of the ‘cellular component’ related genes, most up-regulated DEGs were involved in the ribosome and ribonucleoprotein complex, while most down-regulated DEGs were involved in photosystem II oxygen-evolving complex, oxidoreductase complex, and thylakoid membrane. Meanwhile, of the ‘molecular function’ related genes, most up-regulated DEGs were involved in structural constituent of ribosome, and most down-regulated DEGs were involved in phosphoglycerate mutase activity (Figure 2).

### KEGG pathway classification

To investigate the biological functions, the KEGG pathways annotated all of the selected DEGs. KEGG pathway enrichment analysis revealed that 263 DEGs in S_CNS *vs.* L_CNS, including 152 up-regulated and 111 down-regulated DEGs were respectively assigned with top 20 KEGG pathways. These pathways were subsequently enriched with *P* < 0.01 and are highly significant. We removed pathways in connection to human diseases. Four up-regulated KEGG pathways were significantly enriched, including ribosome, oxidative phosphorylation, antigen processing presentation, and cardiac muscle contraction. Three down-regulated KEGG pathways were significantly enriched including, glyoxylate dicarboxylate metabolism, glycine-serine and threonine metabolism and oxidative phosphorylation (Table 3).

**Table 3:** Enrichment of KEGG pathways of the differentially expressed genes (DEGs), up and down regulated from CNS among S_CNS *vs.* L_CNS

Group	NO.	Term	ID	Corrected P-Value
S_CNS *vs.* L_CNS Up DEGs	1	Ribosome	ko03010	1.13E-06
	2	Oxidative phosphorylation	ko00190	1.51E-06
	3	Antigen processing and presentation	ko04612	0.001918612
	4	Cardiac muscle contraction	ko04260	0.00140975
S_CNS *vs.* L_CNS Down DEGs	1	Glyoxylate and dicarboxylate metabolism	ko00630	0.00639973
	2	Glycine, serine and threonine metabolism	ko00260	0.007731045
	3	Oxidative phosphorylation	ko00190	0.007731045

### Validation of RNA-Sequence data using qRT-PCR

**Figure 3:**
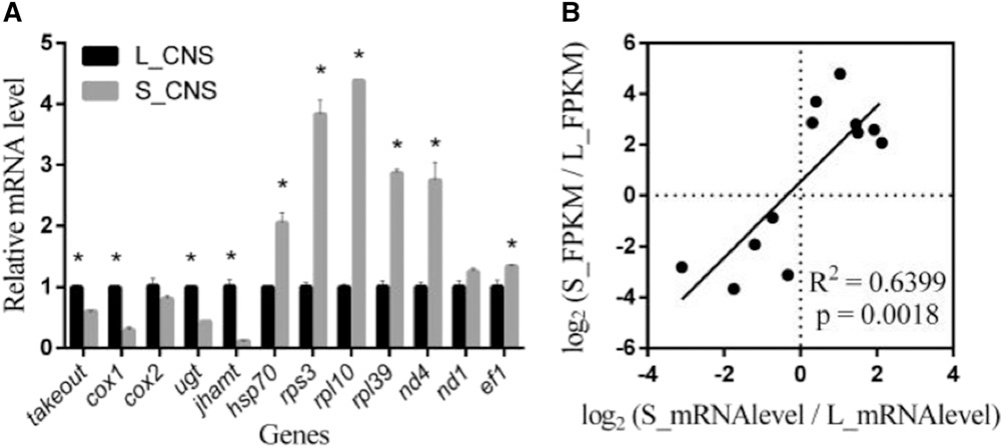
(A) Relative mRNA level of differentially expressed genes from the CNS of *L*. *migratoria* under long and short photoperiods detected by qRT-PCR. (B) Scatter plot of total transcript level measured by real-time PCR (log_2_ -transformed) *vs.* estimation from RNA-Seq. Three technical replicates from each treatment were performed, with *ef-1* gene used as internal control. * Indicates an error probability of *P* < 0.05 using Student’s *t*-test.

In the present study, we confirmed the consistency of RNA sequencing data by qRT-PCR. Twelve DEGs were selected from CNS of *L. migratoria* under both long and short photoperiods. The qRT-PCR result showed that gene expression of *takeout*, *cox1*, *cox2*, *ugt*, and *jhamt* were significantly lower in S_CNS than L_CNS. While gene expression of *hsp70*, *rps3*, *rpl10*, *rpl39*, *nd4*, *nd1* and *ef1* were significantly higher in S_CNS than L_CNS (Figure 3A). Scattered plots of DEGs mRNA level *vs.* FPKM results demonstrated (Figure 3B) consistency between RNA sequencing data and qRT-PCR (R^2^ = 0.6399; *P* < 0.0018). The expression level of RNA-Seq data for each gene showed similar expression profile in comparison with the results of qRT-PCR.

### Function identification of Lm-takeout

**Figure 4:**
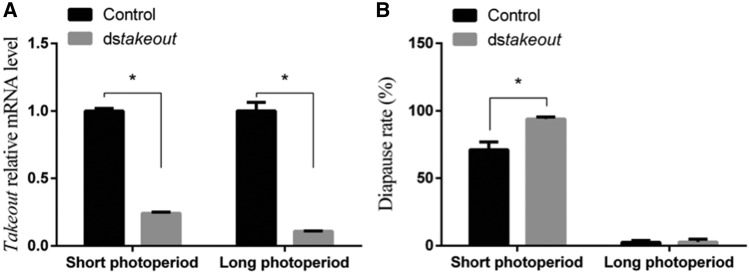
The adult female CNS samples were collected simultaneously two hours after lights on (long day) and lights off (short day). (A) *takeout* expression level in ds*takeout* treatment and control. (B) Diapause rate of ds*takeout* treatment and control under long and short photoperiods in *L*. *migratoria*.

Several genes have been targeted and functionally analyzed for their critical role in insect biology. The *takeout* gene was discovered as a circadian-regulated gene and has similarity with juvenile hormone binding proteins (JHBPs) (So *et al.* 2000). In this study, we selected the *Lm-takeout* gene to explore its role in the maternal effect on egg diapause of *L. migratoria* under long and short photoperiods. The *Lm-takeout* gene sequence was first analyzed by NCBI blast. The full length of *Lm-takeout* gene coding sequence was 771bp, which can encode a protein with 256 amino acids. It showed 49% of identity and 67% of coverage with *takeout* of *Zootermopsis nevadensis*. Similarity to Juvenile hormone binding protein (JHBP) was found at 31-250 aa of *Lm-takeout*. JHBP regulates embryogenesis and reproductive maturation in the adult insect. To verify the function of a ds*takeout* gene on regulating locust diapause, dsRNA of *Lm-takeout* was synthesized and subsequently injected into female adults of *L. migratoria* under both long and short photoperiods. The result showed that the mRNA level of *Lm-takeout* gene was significantly lower under long and short photoperiods as compared to control (Figure 4A). This indicated that the RNAi efficiency was satisfactory. Furthermore, RNAi maternal diapause rate was significantly increased in ds*takeout* treatment under short photoperiod (94.4%) as compared to the control (71.1%) (*P* < 0.003), whereas there was no significant difference between treatment and control under long photoperiod (Figure 4B).

## Discussion

Photoperiod is one of the most critical factors that can affect maternal embryonic diapause in various insect pests (Ponnuvel *et al.* 2015). Previous studies in *Bombyx mori* showed that embryonic diapause is *trans*-generationally induced by maternal photoperiod (Chen *et al.* 2017). Similarly, maternal locusts can be respond to short photoperiod conditions by influencing offspring egg diapause (Wardhaugh 1980; Hao *et al.* 2017).

In the present study, the transcriptomes from CNS of *L. migratoria* induced by both short and long photoperiods were analyzed to determine the differently expressed genes (DEGs) related to maternal egg diapause.

### Differentially expressed genes (DEGs) and pathways associated with diapause and FOXO signaling pathway

Diapause is a complex phenomenon where the FOXO emerges as a prime candidate for activating many of the diverse physiological pathways in diapause induction (Sim *et al.* 2015). Similarly, FOXO plays an essential role in diapause of nematodes and mosquitos (Calixto and Jara 2012; Sim and Denlinger 2008). Growth factors and insulin signaling pathways inhibit FOXO activity, whereas nutrient depletion and a plethora of reactive oxygen species (ROS)-induced post-translational modifications stimulate FOXO activities (Jovanović‐Galović *et al.* 2007; Almeida 2011; Chang *et al.* 2019). A previous study suggested that FOXO, a downstream protein in the insulin-signaling pathway, led to insect diapause (Sim and Denlinger 2008). In the current study, a total of 610 DEGs were obtained in *L. migratoria* CNS by exposing maternal locusts to different photoperiods. Further analysis, including GO and KEGG enrichments, were performed to screen the DEGs related to diapause induction. Results showed that the expression profiles of the DEGs we found were consistent with previous studies on diapause regulation (Table S3).

### Involvement of ribosomal proteins

Ribosomal proteins have numerous primary functions. They serve as the site of biological synthesis with regard to protein rearrangement and enzyme biosynthesis, thus initiating significant changes in the body. S6 ribosome protein has been reported to play role in the upstream pathways associated with diapause induction (Cristofano *et al.* 1998; Qi *et al.* 2015; Wool 1993; Huang *et al.* 2008). Similarly, another candidate ribosome, *RpS3a*, is expressed in ovarian diapause of mosquito, *Culex pipiens* (Kim *et al.* 2010). In our study, the ribosomes, including RPLP0, RPS23, RP-SAe, RPS29, RPS3A, RPL6, RPL8, RPS3, RPL10 and RPL39 were significantly up-regulated in the samples treated under short photoperiod (Table 3). However, with regard to treatments of long photoperiod, no active ribosomes were detected. The expressions of ribosomes were also confirmed by the qRT-PCR (Figure 3; Table S2). On the other hand, the GO classification also revealed the DEGs related to ribosomes. During the diapause induced condition, 39 GO terms in biological process (BP), 73 in cellular components (CC), and 37 molecular functions (MF) were assigned to ribosome biogenesis, ribonucleoprotein complex biogenesis, ribosome, ribonucleoprotein complex and structural constituent of ribosome pathways. These were all found to be significantly up regulated (Figure 2). This indicates that the ribosomes play a potentially important role in diapause induction of *L. migratoria*.

### DEGs and various pathways induced

Several metabolic signaling pathways were induced by treatments under short photoperiod (Table S2), including oxidative phosphorylation (ko00190), FOXO signaling pathway (ko04068), and glycolysis / gluconeogenesis (ko0001). Oxidative phosphorylation related genes, including *cox1*, *cox2*, *cox3*, *nd1*, and *nd4* were found significantly different in S_CNS *vs.* L_CNS (Table S2). Down regulated cytochrome c oxidase is another important biological process that mediates photo-biomodulation and is active in neurons, which terminate the larval diapause period in the bamboo borer, *Omphisa fuscidentalis* (Wong-Riley *et al.* 2005; Singtripop *et al.* 2007). Similarly, *cox1* gene initiates in the brain and is involved in pupal diapause termination in the hornworm, *Agrius convolvuli* (Uno *et al.* 2004). Moreover, cytochrome c oxidase subunit 2 (*cox2*) is involved in diapause of *C. pipiens* (Robich *et al.* 2007). Cytochrome oxidase’s expressions were confirmed through qRT-PCR in the current study. The mRNA levels of *cox1* and *cox2* were down-regulated in CNS samples under short photoperiod. In contrast, the oxidative phosphorylation genes *nd4* and *nd1* were up-regulated in CNS samples under short photoperiod (Figure 3). Thus, the oxidative phosphorylation may be an important component of maternal diapause induction of *L. migratoria*.

Three DEGs, including *pten*, *catalase* (*cat*) and *BNiP3* were detected in the FOXO signaling pathway. The *pten*, being the upstream of the FOXO, has been reported essential for sperm maturation and fertility in different species (Rouault *et al.* 1999; Xu *et al.* 2014), but its role in insect diapause regulation is still unclear. In this study, *pten* was upregulated in S_CNS as compared to L_CNS (Table S2). The presence of *pten* in short photoperiod treated CNS may ultimately have activated the FOXO signaling pathway in *L. migratoria*. Similarly, downstream of *catalase* in the FOXO signaling pathway and its essential role has been reported to encode antioxidant component and promote the diapause and ovaries protection in mosquito, *C. pipiens* (Sim and Denlinger 2011). *PTEN* inhibited the activity of Akt and reduced the FOXO phosphorylation, which may have prevented the down-regulating of *catalase*. Although *cat* was also regulated by the ROS (Zrelli *et al.* 2011). In our study, the *catalase* was down regulated in S_CNS compared to L_CNS (Table S2).

Other DEGs involved in metabolic pathways, such as glycolysis/ gluconeogenesis and TCA cycle, have also been found to be expressed in a rhythmic manner (Wijnen and Young 2006; Kohsaka and Bass 2007). In this study, glycolysis/ gluconeogenesis and TCA cycle pathways were activated and subsequently induced reactive oxygen species (ROS) under a short photoperiod. A previous study has described that the buildup of ROS could damage lipids, DNA, RNA, and proteins that would not be favorable to growth and development (Kidokoro *et al.* 2006). This study indicated that the DEGs involved in glycolysis/ gluconeogenesis are essential for photoperiodic induced diapause of *L. migratoria*.

### DEGs related pathways and their functions

*jhamt* (juvenile hormone acid O methyltransferase) is specific DEG known for its critical and dominant role in JH biosynthesis, which is associated with insulin signaling pathways (Shinoda and Itoyama 2003; Van Ekert *et al.* 2014). In this study, the *jhamt* down-regulated in S_CNS with 0.11880 fold of that in L_CNS transcripts (Table S4) and its expression level is shown through qRT-PCR analysis (Figure 3 A). This indicates that the JH biosynthesis must decreasing in light of the fact that the insulin promotes the JH biosynthesis (Sim and Denlinger 2013; Tu *et al.* 2015). A decreasing trend of JH synthesis indicates that the insulin signaling pathway has been inhibited and promotes the activation of FOXO.

Another super family of enzyme is UDP-glycosyltransferases (UGTs), which determines the allocation of glycosyl residues by activating nucleotide sugars to acceptor molecules. Acceptors regulate the bioactivity, solubility and transport throughout the organism. UGTs play an important role in insecticide resistance and detoxification of plant allelochemicals, however study pertaining to their application in insects is limited (Wang *et al.* 1999). In post-diapause of *L. migratoria*, the *ugt* was down-regulated and had an important role in negative cell morphological changes and maintenance (Hao *et al.* 2017). In the current study, the *ugt* annotated to retinol metabolism pathway (ko00830) showed significantly down-regulated expression in S_CNS as compared to L_CNS (Table S2). This indicated that the *ugt* might be induced by short photoperiod. In addition, some other enriched pathways, related to up and down regulated DEGs, were also identified during the diapause induced conditions including glucagon signaling pathway with ADCY2 gene, Hippo signaling pathway with *Yki* gene, Synaptic vesicle cycle, Peroxisome and Biosynthesis of secondary metabolites pathways etc. (Table S2). DEGs, such as cytochrome P450, Ras-related protein Rab-9B etc, were also found in S_CNS as compared to L_CNS, suggesting individual expressions may be involved in diapause induction of *L. migratoria*. However these connections still need to be further researched.

### Heat shock proteins (HSPs)

Insect HSPs may be the most important subcellular component of the stress response in different organisms during the development phase in response to extreme temperature, hypoxia/anoxia, starvation and individual functions of particular proteins within the HSP family (King and MacRae 2015). The response of HSPs to storage, protein maintenance and protein folding stress tolerance has been previously reported in many insects (Chen *et al.* 2006; Li *et al.* 2007; Rinehart *et al.* 2007; Tungjitwitayakul *et al.* 2008). Heat-shock protein *shp70* was described as up-regulated in embryogenesis egg diapause stage of silkworm, *B. mori*, and in overwintring diapause of related insect species (Rinehart *et al.* 2007; Ponnuvel *et al.* 2010). In the present study, HSPs involved in signaling pathways, such as hsp70 in MAPK signaling pathway (ko04010), and *hsp90*A in PI3K-Akt signaling pathway (ko04151) were up-regulated in S_CNS treated samples of *L. migratoria* (Table S2). Comparing our results and findings of mRNA transcripts indicated that the *hsp70* and *hsp90*A may play a specific role in diapause induction of *L. migratoria*.

### Lm-takeout, a circadian gene regulate maternal diapause

The *takeout* gene, primarily reported in the fruit fly, *Drosophila melanogaster*, was found to be regulated in the circadian rhythm, which plays important role in regulation of feeding behavior, longevity, starvation response, sex specificity, fertility and other related functions in certain insect species (So *et al.* 2000; Fujikawa *et al.* 2006; Meunier *et al.* 2007; Lazareva *et al.* 2007; Chamseddin *et al.* 2012). Our current transcriptomic analysis revealed the expression of *Lm-takeout* at same circadian rhythmic pattern in S_CNS *vs.* L CNS by RNA-Seq. The mRNA level of *Lm-takeout* in L_CNS was 1.88-fold to that of S_CNS. qRT-PCR also confirmed the validity of the relative expression of *Lm-takeout* in L_CNS with 1.66-fold of that in S_CNS (Figure 3; Table S4). These results indicated that expression of *Lm-takeout* in S_CNS is significantly lower than that of L_CNS. This further suggests that the diapause induction in maternal locust seems to be negatively regulated by *Lm-takeout*. To confirm this hypothesis, RNAi was performed to identify the diapause regulation function of *Lm-takeout*. Results showed that RNAi maternal *Lm-takeout* gene significantly promoted offspring diapause in *L. migratoria* (Figure 4). Previously, *takeout* has been linked to the circadian clock, being an output gene, which is regulated by *Pdp1*, and was found to be involved in locomotor activity. Additionally, *Pdp1* served as a transcriptional regulator in the brain or fat body of flies (Sarov-Blat *et al.* 2000; Benito *et al.* 2010). In *Riptortus pedestris*, the *period* and *cycle* genes of the circadian clock under different photoperiods regulated the ovarian diapause (Emerson *et al.* 2009). Similarly, the circadian clock related genes, such as *period*, *timeless* and *chrptochrom2*, have also been reported in photoperiodic diapause study of *Culex pipiens* (Meuti *et al.* 2015). This strongly suggests that the circadian clock related genes could serve as an input module or transmitter to transfer the light signals to the insulin signaling pathway and finally regulate diapause in *Culex pipiens* (Sim and Denlinger 2013). Thus, the *takeout* gene was considered to be a downstream effector of the circadian clock function in *Culex pipiens* (Sim *et al.* 2015). This indicated that the *Lm-takeout* gene of *L. migratoria* might also be bridging between the circadian clock and downstream insulin-signaling pathway to lead the offspring egg diapause.

**Figure 5:**
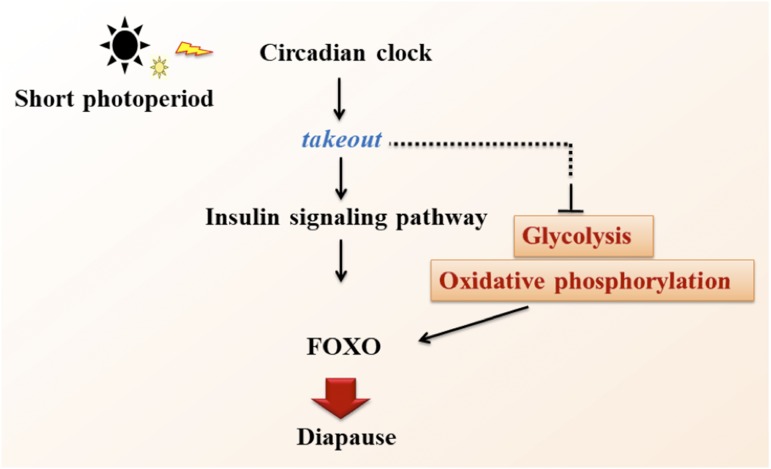
Hypothetical pathway of circadian clock where the *takeout* gene transmits photoperiod related signals to maternally affect on egg diapause induction of *L*. *migratoria*.

The transcriptome analysis showed that the FOXO signaling pathway is the most photoperiodic diapause-related pathway in CNS. Most of the DEGs are involved or related to the FOXO signaling pathway. We identified that the *Lm-takeout* gene plays a significant role in diapause regulation of *L. migratoria*. Although we do not have direct evidence that *Lm-takeout* regulates diapause induction through FOXO signaling pathway, we propose that the simultaneous screening of DEGs in S_CNS *vs.* L_CNS was not accidental, rather linked intrinsically. Our results suggest a model in which photoperiod might affect the FOXO signaling pathway through the maternal *Lm-takeout* by producing environmental signals transmitted to the next generation for inducing the egg diapause in locusts (Figure 5).

### Conclusions

In the present study, a CNS based transcriptome database of *L. migratoria* under long and short photoperiods was created. We have extended new insights into the genomics of CNS of *L. migratoria*. Our results added a useful reference to further highlight the comparative information on molecular mechanisms and diapause induction of *L. migratoria*. Based on the transcriptome analysis and DEGs related findings, our results will contribute significantly to unveil the diapause-specific candidate genes and pathways related to FOXO signaling pathway. From among the identified DEGs, the *Lm-takeout* gene was selected and confirmed to be involved in maternal egg diapause of *L. migratoria* because of its association with circadian clock. This work provides valuable data with regard to further investigation on the molecular mechanisms underlying diapause induction.
